# On the viability of implantable electrodes for the natural control of artificial limbs: Review and discussion

**DOI:** 10.1186/1475-925X-11-33

**Published:** 2012-06-20

**Authors:** Max Ortiz-Catalan, Rickard Brånemark, Bo Håkansson, Jean Delbeke

**Affiliations:** 1 Department of Signals and Systems, Biomedical Engineering Division, Chalmers University of Technology, Göteborg, Sweden; 2 Centre of Orthopaedic Osseointegration, Department of Orthopaedics, Sahlgrenska University Hospital, Göteborg, Sweden; 3 School of Medicine (MD), Institute of Neuroscience (SSS/IoNS/COSY), Université catholique de Louvain, Brussels, Belgium

**Keywords:** Electrodes, Biopotential electrodes, Implantable electrodes, Neural interfaces, Artificial limbs, Prosthetic control

## Abstract

The control of robotic prostheses based on pattern recognition algorithms is a widely studied subject that has shown promising results in acute experiments. The long-term implementation of this technology, however, has not yet been achieved due to practical issues that can be mainly attributed to the use of surface electrodes and their highly environmental dependency. This paper describes several implantable electrodes and discusses them as a solution for the natural control of artificial limbs. In this context “natural” is defined as producing control over limb movement analogous to that of an intact physiological system. This includes coordinated and simultaneous movements of different degrees of freedom. It also implies that the input signals must come from nerves or muscles that were originally meant to produce the intended movement and that feedback is perceived as originating in the missing limb without requiring burdensome levels of concentration. After scrutinizing different electrode designs and their clinical implementation, we concluded that the epimysial and cuff electrodes are currently promising candidates to achieving a long-term stable and natural control of robotic prosthetics, provided that communication from the electrodes to the outside of the body is guaranteed.

## Introduction

Although myoelectric prostheses were devised in the late 1940s [1] and have been clinically implemented since the 1960s [2], they are still a long way from the functionality of their biological counterpart. Moreover, myoelectric control strategies used back then, such as the activation of a group of muscles (e.g. flexors) to control the direction of a single degree of freedom at the time (e.g. hand close), are presently the most commonly used.

Prosthetic hardware is now considerably more advanced than the control strategies needed to command it. Sophisticated prostheses such as the SmartHand [3] provide more degrees of freedom than a patient is able to control naturally. Furthermore, sensors embedded in the prosthesis can provide valuable feedback information that currently has no stable means of being naturally transmitted to the patient [4].

In this context, “natural” is defined as producing control in the same way as an intact physiological system. This means coordinated and simultaneous movements of different degrees of freedom. It also implies that the input signals must come from nerves or muscles that were originally meant to produce the intended movement (physiologically appropriate). Natural control requires that feedback is perceived as originating in the missing limb, without overwhelming concentration on the part of the user. Natural control is a fundamental feature that is greatly appreciated by patients [5].

Now that prosthetic hardware is available, there are two straightforward strategies for improving the current state-of-art of robotic prosthetics. 

1. **Signal processing and control algorithms**. This includes the development of improved signal processing, pattern recognition and control algorithms that compensate for the lack and instability of the bio-signals.

2. **Signal acquisition**. This improves the way bio-signals are retrieved by the development and optimization of electrodes, as well as optimal source selection and enhancement.

Despite the extensive studies that have been conducted on signal processing techniques and pattern recognition algorithms (PRAs), this approach appears to have reached a steady state where several PRAs have been found to reach similar levels of accuracy [6]. In this work we discuss how the use of implantable electrodes could resolve the practical problems that are currently preventing the long-term implementation of an advanced prosthetic control based on PRAs. The viability of these electrodes was analyzed by reviewing their current state of the art, but more importantly, their clinical implementation in the peripheral nervous system (PNS). The electrode technology itself was previously reviewed in 2004 by Navarro *et al.*[7], and in 2008 by Cogan with the emphasis on microelectrodes for stimulation [8]. A review of intrafascicular electrodes with focus on the central nervous system (CNS) is given by Cheung in [9]. A recent and extensive review in myoelectric control is given by Scheme and Englehart in [10].

In this work, we aim to summarize and provide a general framework with which a target prosthetic implementation (in our case, bone-anchored prostheses) can find a specific electrode solution that matches the needs of the patient while limiting risk, providing the longest-term implementation and, more importantly, a more natural prosthetic limb replacement.

## Review

### Electrode Types and Classification

In order to avoid the ambiguity in the word “electrode”, an electrode is hereinafter regarded as a device including a fixation structure forming the electrode body and one or more contacts. There are several types of biopotential electrode, but no standardized classification is available. Authors have classified them by function, material, size and geometry amongst other features. When it comes to prosthetic control, electrodes can be divided into two general categories: **Surface Electrodes (SEs):** A non-invasive type located over the skin which renders a general motor unit action potential, or spatial characterization of the electric potential distribution. **Implantable Electrodes (IEs):** An invasive type that provides more localized measurements.

The SEs are currently the most commonly used electrodes in myoelectric prostheses. Lake and Miguelez classified them as [11]: **Non-Remote:** The pre-amplifier and electrode contacts are housed together. **Remote:** The pre-amplifier is placed in a different container than the electrode contacts.

These electrodes are normally used in a bipolar configuration for a differential measurement and are made of biocompatible metals such as stainless steel or PtIr. Implantable electrodes, on the other hand, are more diverse in terms of configuration, shape and material. The following classification of IEs was presented by Grill in [12]: **Muscle-Based Electrodes (MBEs):**

· *Epimysial*

· *Intramuscular*

Nerve-Based Electrodes (NBEs):

· *Epineural and Cuff*

· *Intraneural or Intrafascicular*

Merletti and Parker described several electrodes for recording EMG and ENG [13] that are not necessarily designed to be permanently implanted and can be seen as a sub-classification of the MBEs and NBEs. 

· *Needle Electrodes:* Operate by muscle and nerve penetration.

· *Micro-electrodes Array:* Array of needle electrodes with equal or different lengths.

**Figure 1:**
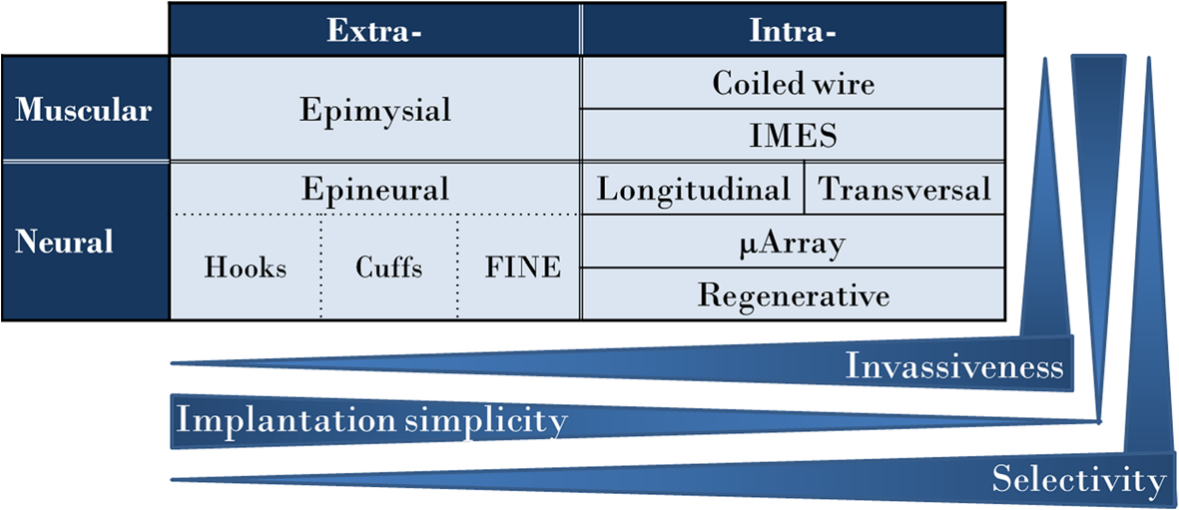
**Implantable electrodes (IEs) for prosthetic control.** This classification uses 4 categories taking account of the target and the location within the target: 1) extra-muscular, 2) intra-muscular, 3) extra-neural, 4) intra-neural. The invasiveness is proportional to selectivity and inverse to the simplicity of the implantation. IMES stands for Implantable Myoelectric Sensors.

Summarizing all the different implantable electrodes and the presented classifications, they could all be divided into 4 categories taking account of the target and the location within the target. The target is neural or muscular and the location is related to invasiveness into the target, see Figure 1. The following is a non-exhaustive list of promising IEs for prosthetic control in the proposed classification. These were also the electrodes investigated in this study. **1.- Extra-muscular**

· *Epimysial*

2.- Intra-muscular

· *Coiled Wire*

· *Radio Frequency BIOnic Neuron* (RF BION)

· *Implantable Myoelectric Sensors* (IMES)

3.- Extra-neural

· *Cuff*

· *The Flat Interface Nerve Electrode* (FINE)

4.- Intra-neural

· *Longitudinal Intrafascicular Electrode (LIFE)*

· *Transverse Intrafascicular Multichannel Electrode (TIME)*

· *Micro-Electrode Arrays (MEAs)*

· *Regenerative Electrodes*

Although all these electrodes could be used for recording and stimulation, the purpose dictates their optimal design and separates them. The amount of charge that can be safely induced during stimulation is limited by the electrode contact area. Higher currents can be passed through a larger surface. At the same time, the impedance is reduced, as well as the requirements for the stimulator supply potential, which in turns prolongs battery life. On the other hand, a small surface area typically yields larger signals, making them easier to record. In this work both clinical applications are considered when describing the different electrodes.

All human-machine interfaces, especially implantable electrodes, must be passively and actively biocompatible [12]. **Passive biocompatibility** relates to the tissue reaction to the composition, shape and mechanical properties of the electrode materials. **Active biocompatibility** relates to the performance of the device during operation. 

· In the event of stimulation, the delivered current should not damage the tissue or cause chemical reactions that form toxic components around the electrode.

· Electrode position and signal delivery should be kept constant under dynamic conditions of muscular movement in order to avoid tissue injuries.

Since the ultimate goal of most IEs is to be chronically implanted, they are manufactured with long-term biocompatible materials to ensure passive biocompatibility. Active biocompatibility, however, depends strongly on the electrode design, location and intended use. Mechanical stress caused during muscular contractions and thereby limb movements must be considered for all IEs. An update on the biocompatibility of microelectrodes is given in [14] and a more detailed review of the brain tissue response can be found in [15].

The electrode materials determine their electrical behavior in contact with the electrolyte and allow another general classification according to the way the current flows in the electrode-electrolyte interface (EEI) [16]. **Perfectly Polarizable:** The properties of these electrodes, such as electrode potential and conductivity, change due the passage of electric current. Theoretically, no actual charge crosses the EEI when a current is applied and the present current is therefore a displacement current. These electrodes behave like capacitors. **Perfectly Non-Polarizable:** The current flows freely between the EEI and therefore requires no energy to make the transition.

No electrode can be fabricated to be completely polarizable or non-polarizable, but these ideals can be closely approached. Electrode contacts made with noble metals are considered to be close to perfectly polarizable [16] and, as these metals are normally biocompatible, IEs for long-term implantation are normally the perfectly polarizable type. The Ag/AgCl contacts are an example of a non-polarizable electrode which cannot be implanted due to biocompatibility issues. Coatings such as PEDOT (described later), however, represent alternative attempts to make implantable non-polarizable contacts.

### Muscle-based Electrodes (MBEs)

MBEs have a well-established record of safety and efficacy, but they can damage muscle fibers that surround them due to mechanical stress. Epimysial and coiled wire (intramuscular) electrodes are considered to have good selectivity and a low risk of damaging nerves in their surroundings [12].

#### Epimysial Electrodes

These extra-muscular electrodes are sewn onto the epimysium, causing less damage to the muscle fibers than the intramuscular electrodes [17] and, since they are exposed to less mechanical stress than the intramuscular type, their life expectancy is longer. It has been shown that, after encapsulation, the epimysial electrode incorporates into the fascia and does not move with the muscle [18]. It was also observed that the electrode was loose within the encapsulation pocket, which presumably reduces some of the mechanical stress.

**Figure 2:**
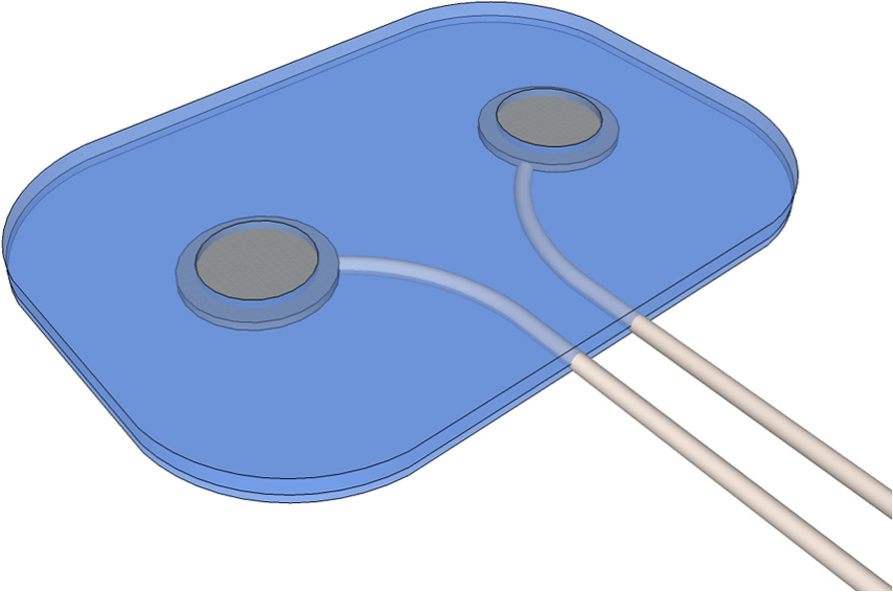
**A bipolar epimysial electrode.** Illustration of a bipolar epimysial electrode with two PtIr discs mounted and covered by silicon sheets in which round windows allow the exposure of the PtIr.

Epimysial electrodes have a good record of stability and have been widely used in humans; they are more commonly found in neuroprostheses to perform functional electrical stimulation (FES) [19-22]. These electrodes are normally PtIr discs mounted on a silicone backing in a bipolar configuration, see Figure 2. In a study in 2005, some problems were reported while placing the electrodes in the optimal position during surgery. The electrode placement was performed using stimulation to find the best motor point, since the patient was under general anesthesia. One electrode, however, had to be reinstalled in a subsequent operation, resulting in an increase in the signal by an order of magnitude [19]. These issues exemplify some of the drawbacks when using implantable electrodes. The recording electrode in this experiment was in a bipolar configuration consisting of discs with a 4 mm diameter placed 10 mm apart. A monopolar configuration has also been successfully employed in lower-extremity neuroprostheses (Pt10Ir disks of 10 *mm*^2^) [20]. The lifetime of these electrodes was estimated to be 4 years, since they are exposed to greater mechanical stress. In upper limb neuroprostheses, however, epimysial electrodes have been reported to remain functional for more than 16 years [22].

#### Intramuscular Electrodes

**Figure 3:**
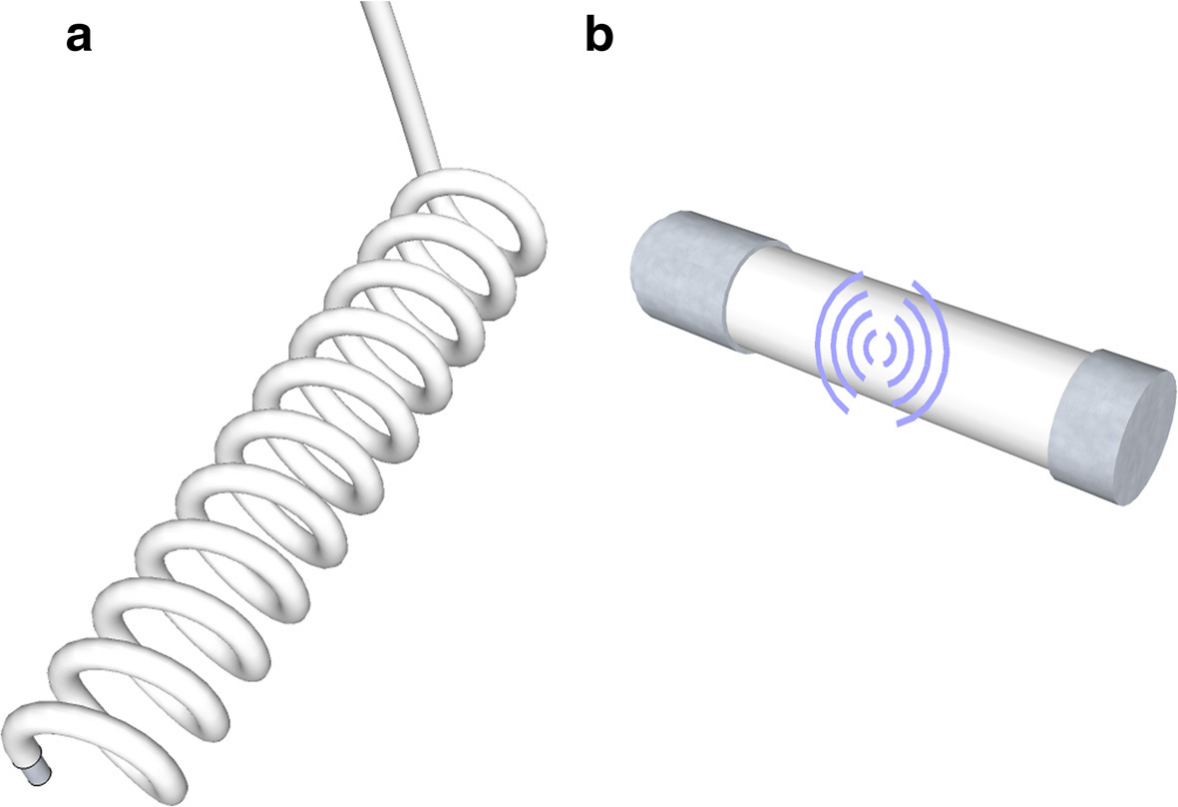
**Intra-muscular electrodes.** Illustrations of (a) a coiled wire electrode where the exposed tip is the sensing part, and (b) an implantable myoelectric sensor where the sensing part is the electrode caps.

A common type of intramuscular electrode is the coiled wire that can be placed percutaneously or be fully implanted [12]. The spiral allows certain movement that is necessary during muscle contractions, Figure 3(a). A single coiled wire could be used in a monopolar configuration with a reference located outside the muscle, or in a bipolar configuration together with another coiled wire electrode, both inside the muscle.

More sophisticated devices in this category include the RF BION (Radio Frequency BIOnic Neuron) [23] and the Implantable Myoelectric Sensors (IMES) using the BION package [24]. This technology is conceptually a more interesting solution for long-term implementation. Functional and therapeutic electrical stimulation are the main focus of the RF BION, while the IMES are designed for prosthetic control.

These devices can be implanted percutaneously without the need of a surgery, and they are powered, commanded, and addressed, using telemetry from an external coil. In the case of amputees, the coil is placed in the socket of the prosthetic device, and the reading data can be retrieved using reverse telemetry. IMES are small (16.7 mm × 2.4 mm) wireless intramuscular devices with bipolar electrodes that can transmit raw or integrated myoelectric signals (MES). Details of its technical description can be found in [24,25]. An illustration of an IMES is shown in Figure 3(b); the electrode caps are the sensing part, while the embedded electronics are in the center.

This technology has great potential for practical implementation since it eliminates the problems associated with percutaneous wire passage, such as infections, breakage and marsupialization [24]. The IMES have remained fully operational for over 9 months in cats [24], and over 2 years in a rhesus monkey [26]. The feasibility of prosthetic control was demonstrated in the latter experiment where a parallel LDA classifier was used to predict finger movements (flexion/extension of 3 fingers and no movement).

The RF BION has been tested in humans to treat post-stroke shoulder subluxation in hemiplegic subjects and knee osteoarthritis. Clinicians did not report any difficulties during implantation and patients reported negligible pain during and after the procedure. The RF BIONs did not migrate and were tolerated by 12 subjects apart from one who requested its removal [27].

### Nerve-based Electrodes

#### Cuff Electrodes

Cuff electrodes reduce the problems of mechanical stress and displacement that are common in muscle-based electrodes. This reduces the probability of electrode or lead failure [28]. However, in the event of failure, they are possible to retrieve and replace, causing less damage than other more invasive electrode designs, such as the intraneural type.

Muscle length and limb position do not affect readings and stimulations with cuff electrodes. It has been shown that nerve tissue encapsulates the electrodes and acts as a stabilizer, preventing cuff displacements during limb movements [29].

Impedance and stimulation thresholds in these electrodes have shown a high degree of stability over time [29-31]. It has been reported that implanted bipolar cuff electrodes retain functionality over 12 years of stimulating the peroneal nerve in patients with hemiplegia [32]; over 10 years as part of a visual prosthesis in the optic nerve [33]; and up to 7 years stimulating the median and ulnar nerve for pain control [34]. The implantation of these electrodes with stimulation purposes in humans also includes the treatment of obstructive sleep apnea [35] and other neuroprostheses [36].

Although this neural interface has been mostly used with stimulation purposes, it has also shown promising results recording neural activity. In animal experiments, cuff electrodes have been successfully used to measure respiratory output [37] and forces applied to the footpad [38,39]. In humans, feedback for thumb force control was implemented by recording cutaneous slip signals in a quadriplegic patient [40]; in a hemiplegic patient, recordings from the sural nerve were used for footdrop correction [41]. The latter was one of the first studies that demonstrated that natural sensory nerve activity in man can be chronically recorded [42].

Furthermore, cuff electrodes have rendered proof of selectivity in both recordings and stimulations. Single-channel cuff electrodes in a tripolar configuration have been successfully used to discriminate two to three different afferent signals, such as proprioceptive, mechanical and nociceptive [43]. On the stimulation side, it has been shown to be possible to stimulate different nerve fibers depending on the stimulation signal [29,44]. Pulse amplitude modulation and pulse duration modulation techniques have shown nerve selectivity in different configurations [45]. This is relevant to neural feedback which is extremely important for the closed-loop control required in advanced prosthetics.

**Figure 4:**
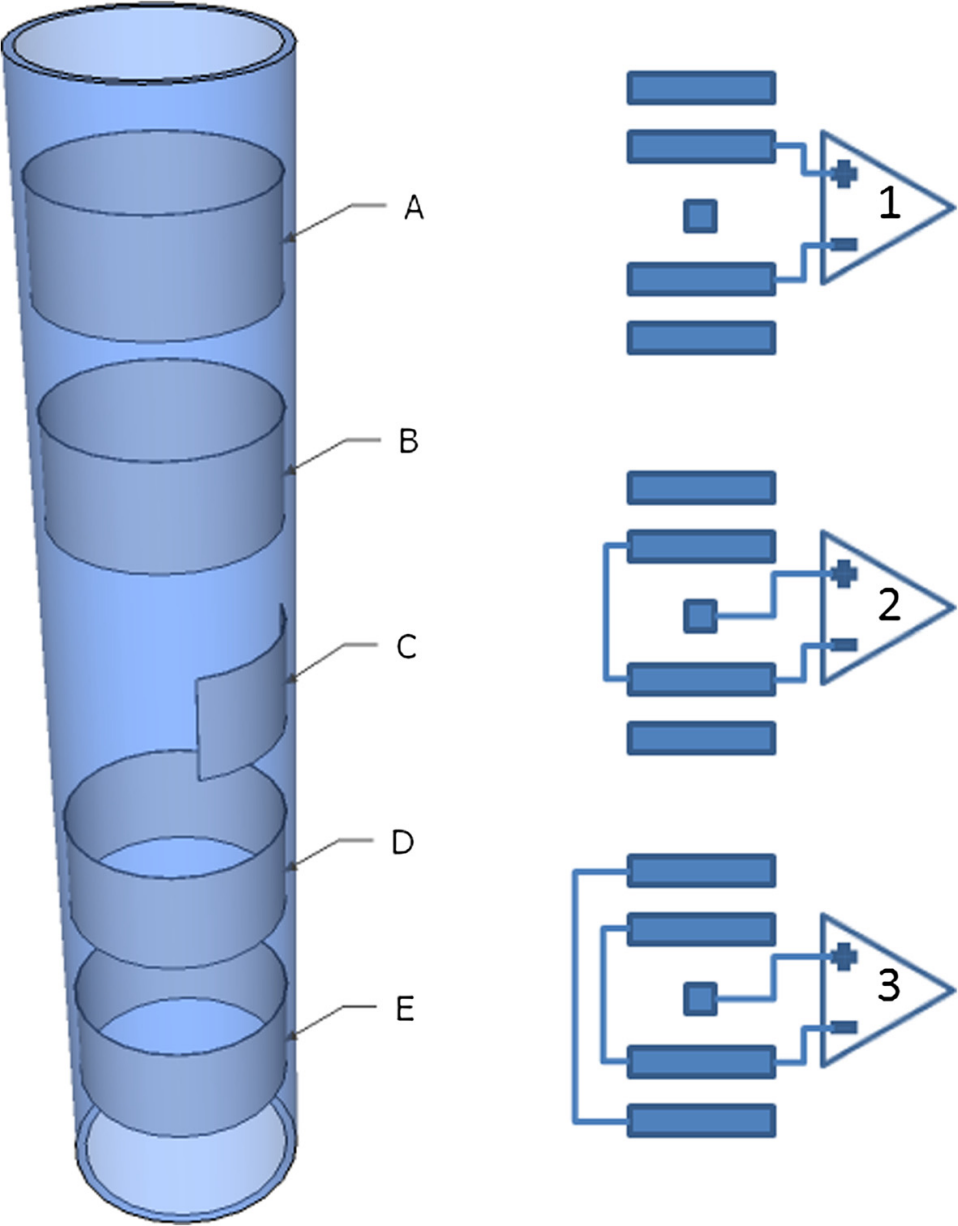
**Cuff electrode.** Cuff electrode with circumferential (A, B, D and E) and discrete (C) contacts with possible differential configurations in bipolar (1), tripolar (2) [46] and short-circuit tripolar (3) [47]. In the latter configuration, the outer electrode pair is short-circuited in order to yield a screening effect [47].

Cuff electrodes usually have circumferential contacts, but discrete contacts (see Figure 4) have proven to be advantageous using steering current to better localize the stimulation [44]. Most of the research for recording purposes has been done using circumferential designs [31,39,40] and little has been reported about the amount of information that can be recorded with discrete contacts. Stimulation, on the other hand, has been very successful with the latter configuration [29,44,45].

A cuff is better than hooks because the local restriction of the extracellular space increases the resistance of the extracellular return path and therefore increases the amplitude of the recorded signals [30,37]. Loeb and Peck have compared several cuff electrode designs and have produced the best results when using an elastic silicon sealed flap [31]. This supports the idea of having a confined space in order to improve readings and stimulations.

Waiting for a stabilization period after implantation is recommended in order to achieve stable readings or thresholds for stimulation. After this period, the cuff is encapsulated by connective tissue and has less variable resistance. A period of around 8 weeks was suggested by Grill and Mortimer [29].

Cuff electrodes have been shown to cause a loss of myelinated fibers inside the cuff of up to 27% and distal to the cuff of 24% soon after implantation. However, these fibers subsequently regenerated and no signs of control or strength losses were found, even though the fibers did not regenerate up to the same diameter [48]. Carp *et al.* conclude that the cuff electrode causes some degree of initial denervation, but that only a modest change in motor unit properties remained after the reinnervation process, finally resulting in very limited damage [49].

The following changes have been reported after cuff electrodes implantation [28]. 

· Loss of function of the innervated muscles

· Increased amount of intraneural connective tissue

· Demyelination of the hosting nerve

· Reduction in the population of larger axons

Possible causes have been suggested including: 

· Surgical trauma to the blood supply or to the nerve itself

· Direct blood contact which is toxic for axons and nerve cells

· Nerve constriction from the cuff during swelling and due to excessive encapsulation.

· Mechanical stress transmitted through leads

**Figure 5:**
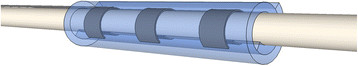
**Spiral cuff electrode.** Illustration of a self-sizing spiral cuff electrode with continuous or ring contacts.

Naples *et al.*[28] found that the self sizing spiral cuff electrode (Figure 5) induces demyelination and axon losses, perineurium thickening, increased intraneural tissue or axonal swelling. To the contrary, in a 4.4-month implantation study with the same electrode in cats, Romero *et al.* concluded that the effects of long-term implantation are negligible [50]. They attributed these discrepancies to the location of the cuff, i.e. a cuff would be more harmful when implanted closer to a joint. The self sizing spiral cuff design is inherently safer than rigid cuffs and more practical in the clinical setting because it can adapt to different nerve diameters [51]. This cuff design has been successfully used in long-term implantations in humans, e.g. [33,52]. However, these electrodes are traditionally hand crafted, which generates a reproducibility issue. Platinum deposition and laser beam activation have been used in a new manufacture method in the hope to solve this problem. This new platinum metalized-silicon rubber has been shown to be as biocompatible as medical graded silicon rubber and plain platinum [53].

A shape memory alloy armature has been suggested as a safe alternative to the self sizing spiral cuff [54]. Hoffer *et al.* have also concluded that even a rigid cuff electrode is safe and stable when properly built and installed [36]. Alternatively, and due to the increasing interest in this technology, micro-electro-mechanical system (MEMS) is currently being exploited to resolve the issue of excessive pressure around the nerves [55].

It has been suggested that most of the mechanical damage is primarily due to surgical handling rather than a long-term implanted cuff. This underlines the importance of proper implantation [51]. The following recommendations have been made [17,31,50]: 

· Keep the surgical intervention time short

· Do not disturb the nerve blood supply (difficult with long cuffs)

· Leave space for the volume of tissue that will form around foreign material

· Implant in a site with relatively small movement. Joints and the surrounding muscle movements can be deleterious

· Limit pulling the nerve to elevate it during cuff wrapping

· Avoid the possibility for cables to impose torque on the hosting nerve

· Leads must be flexible

For optimal recordings, the following conditions should be respected as far as they do not conflict with the previous safety recommendations: 

· A close fitting is necessary to ensure relatively large signals

· The impedance of all the contacts must be low and roughly equal

· A tripolar configuration minimizes the noise induced by myoelectric signals (Figure 4) [46]

· A short-circuit tripolar configuration has a higher signal-to-noise ratio (Figure 4) [56]

· The cuff diameter should be small compared with its length [30]. It has been suggested that the cuff length should be no less than 15 mm or 10 times larger than its inner diameter [57]

#### The Flat Interface Nerve Electrode (FINE)

The main functional issue when it comes to the standard cuff electrodes is their limited access to information from central fascicles. The flat interface nerve electrode (FINE) solves this problem by reshaping the nerve into a flat geometry, thereby forcing all the fascicles close the epineurium [58]. The idea behind this design is to push the natural reshaping capability of the nerve in order to access most of the fascicles without compromising the blood-nerve barrier. This electrode has successfully shown recording selectivity in simulations and animal experiments [59]. It has also been used for stimulation purposes as part of a gait controller in rabbits [60], and in acute experiments in the human femoral nerve [61]. In this first implementation in humans, the FINE proved to achieve selective activation of at least 4 out of 6 muscles. The combination of low invasiveness and high selectivity makes this electrode a promising neural interface, however, its long-term biocompatibility still needs to be proven in humans.

#### Longitudinal Intrafascicular Electrodes (LIFEs)

In 2005, Dhillon and Horch implemented one of the first direct neural feedbacks (position and strength) using an electrode implanted within individual fascicles in severed peripheral nerves [62]. *Longitudinal intrafascicular electrodes* (LIFEs) were used acutely in 6 patients with amputation levels at or below the elbow. Each LIFE was 20-30 cm long, 25 *μ*m diameter and communicated percutaneously. In order to record extrinsic muscles of the hand, 4 to 8 electrodes were implanted in the median nerve (gain of 20,000, band-pass filter 0.3-4 kHz). A “Utah Arm” was used as the artificial limb and a strain gauge and angle sensor provided the feedback that was mapped to the stimulation electrode. More recently, Horch *et al.* demonstrated the opportunity for object discrimination by providing sensations of touch and finger position. This was achieved by implanting LIFE electrodes in the median and ulnar nerves of two trans-radial amputees during two weeks. Again, 25 or 50 *μ*m diameter, 90Pt-10Ir LIFEs were used [63].

**Figure 6:**
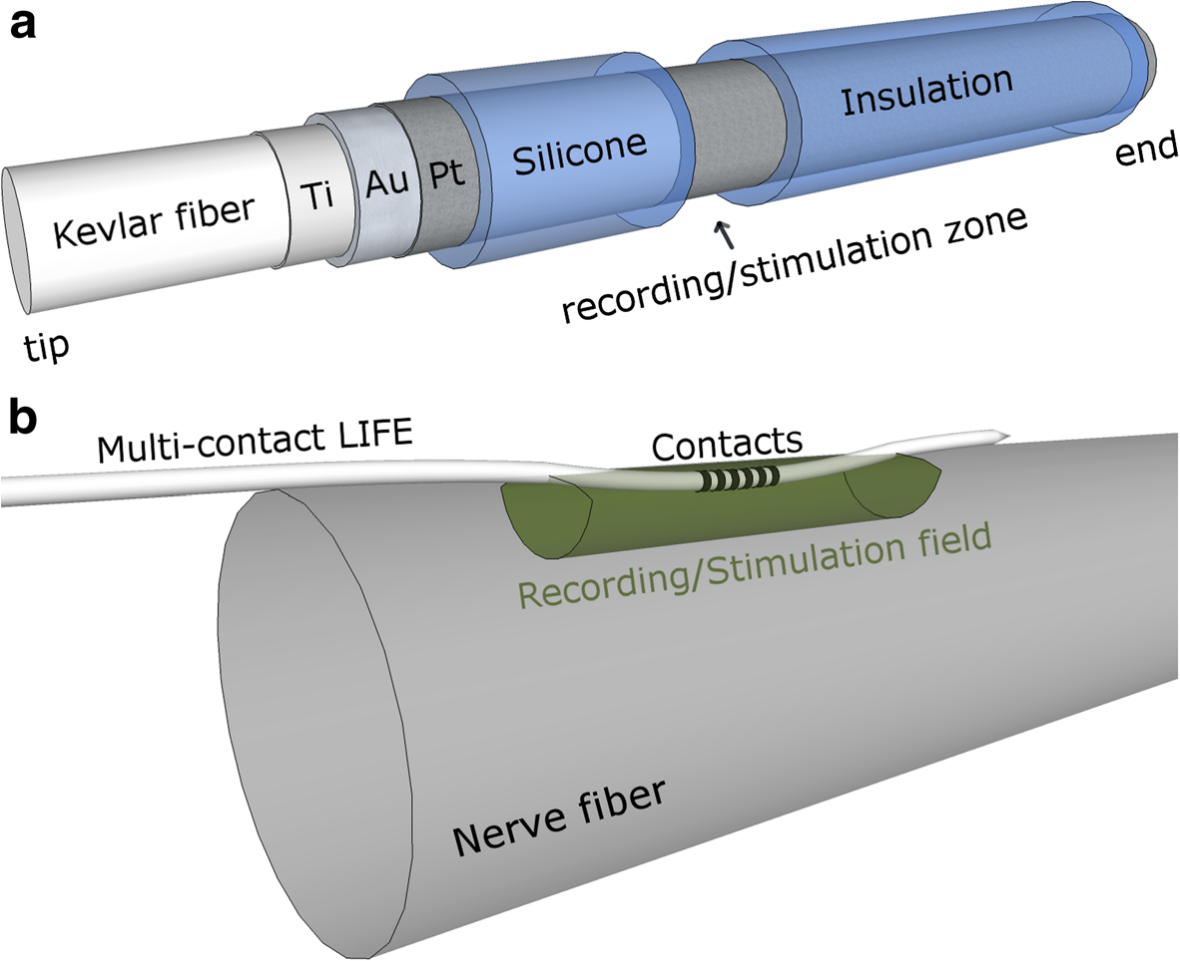
**Intrafascicular electrodes.** Illustrations of (a) a polymer-based intrafascicular electrode (polyLIFE) based in the design from [67] and a multi-contact longitudinal intrafascicular electrode.

Similar *polymer-based longitudinal intrafascicular electrodes* (polyLIFEs) have been chronically implanted in animals and no significant effect on fiber counts, nerve-fiber diameter or myelin thickness were reported [64](Figure 6(a)). Furthermore, it was shown that individual units could be recorded for more than a six-month period in cats using intrafascicular electrodes inside single fascicles [65]. Another polymide-based intrafascicular electrode known as the actuated intraneural (ACTIN) interface that could work cyclically after implantation has been developed using shape memory alloys. This electrode can selectively and independently reposition its active sites inside the nerve in order to improve the electrical connections with the surrounding axons [66].

The LIFEs can have one or more contacts following one another in series, as illustrated in Figure 6(b). These electrodes, such as the transverse intrafascicular multichannel electrode (TIME), can be positioned with different orientations inside the fascicles in order to achieve more selectivity [68]. The SELINE, a self opening intrafascicular neural interface, is the next generation along this line by combining LIFE and TIME spatial features [69]. This design includes wing-like structures that improve anchorage, and at the same time creates a 3D distribution that theoretically increases selectivity.

Probably one of the most interesting clinical experiments to date is the use of 4 intrafascicular longitudinal flexible multielectrodes (tf-LIFE) to control a robotic hand [70]. A human patient was implanted with tf-LIFEs for a month and, through pattern recognition algorithms, three different movements were differentiated for the first time [71].

#### Micro-Electrode Arrays (MEAs)

In 2002, Gasson *et al.* implanted a MEA that remained in the median nerve of a healthy human for 96 days [72]. The electrode was made from high purity monocrystalline silicon with platinum-coated active tips. It had 10 x 10 needles with a length of 1.5 mm and 80*μm* diameter (4*μm* at the tip). The perineurium was dissected and the electrode was pneumatically injected into the first available fascicle which was estimated to have a 4 mm diameter. Two PtIr wires were positioned in the fluids surrounding the nerve as electrical reference. The subject did not experience any loss of sensitivity or movement while the electrode was in place and after it was removed. Recording and stimulation were demonstrated in this experiment showing that the MEA can work as a bi-directional interface. However, the information obtained from the recordings was only used in a threshold detection scheme.

The *Utah electrode array* (UEA) is another silicone-based MEA consisting of 100 needle-shaped electrodes of different lengths, a distinctive feature from previous work on MEAs. Each needle is less than half the diameter of a human hair (<80*μm*), and varies in length from 0.5 mm at one end of the array to 1.5 mm at the opposite end [73]. In order to be implanted, the array is literally shot against the nerve using a pneumatic tool, in a procedure similar to [72]. The array is stabilized with a silicon sheath that is wrapped around the nerve and electrode. All the leads went to a percutaneous connector which was the source of several problems and motivated the team to consider a wireless interface. A wireless solution, the Utah Slant Electrode Array/Integrated Neural Interface-Recording version 5 (USEA/INI-R5), has reported functionality for over 9 months in an in-vitro test [74]. The electronics embedded on the backside of the array allows the transmission of signals recorded from the 100 needle electrodes.

The UEA has been used to evoke fatigue-resistant contractions in a force closed-loop controller in felines [75]. In a macaque monkey, a UEA implanted in the motor cortex (M1) was used to decode individual and combined finger movements with classification accuracies over 92% [76]. In a similar model, grasp restoration has been shown possible using a UEA to decode brain activity [77]. Interestingly, the classifier was trained once per week to be used in daily experiments for the rest of the week. This contrasts with traditional pattern recognition using surface electrodes, where the classifier has to be trained for each session. In humans, simple prosthetic control has been demonstrated using a MEA implanted in the motor cortex of a tetraplegic patient [78]. Furthermore, this patient was still able to control a computer cursor after 1000 days of the MEA implantation [79].

High selectivity is a major design advantage of MEAs, however, their capability to discriminate single units has been observed to deteriorate over long periods of time [80]. Chestek *et al.* found a slow but significant (2.4% monthly) decay of the action potentials amplitude during the whole length of the study (over 31 months), although this was not correlated to the classifier performance [81]. The loss of selectivity was overcome by using a threshold detection method instead of well-isolated action potentials from single neurons.

**Figure 7:**
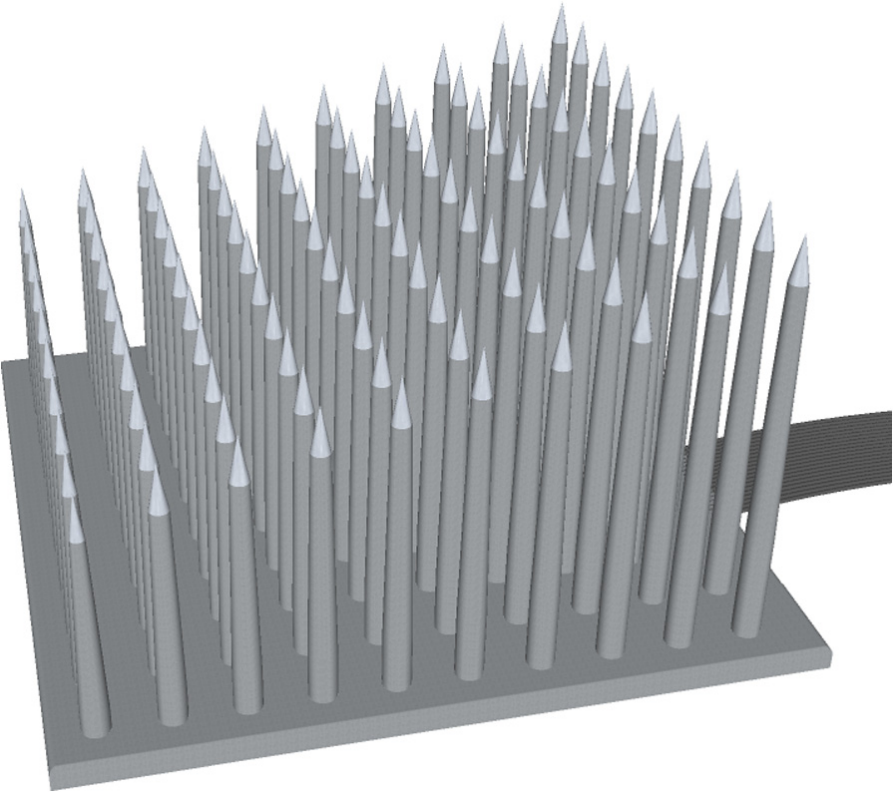
**Micro-electrode array.** Illustration of a micro-electrode array where the tip of each needle is the sensing area. Sketch based in [73].

It is worth noting that most of the research done in MEAs has been in the CNS [76-81], thus the long-term performance of MEA when implanted in the human PNS is yet to be proven. Despite the reported functionality and safety of the MEAs, their implantation compromises the perineurium and the blood-nerve barrier, carrying a significant risk of nerve trauma. This fact, together with a relatively complex implantation, and further stabilization, are the main drawbacks of this interface. An illustration of a MEA (UEA like) is shown in Figure 7.

#### Regenerative electrodes

**Figure 8:**
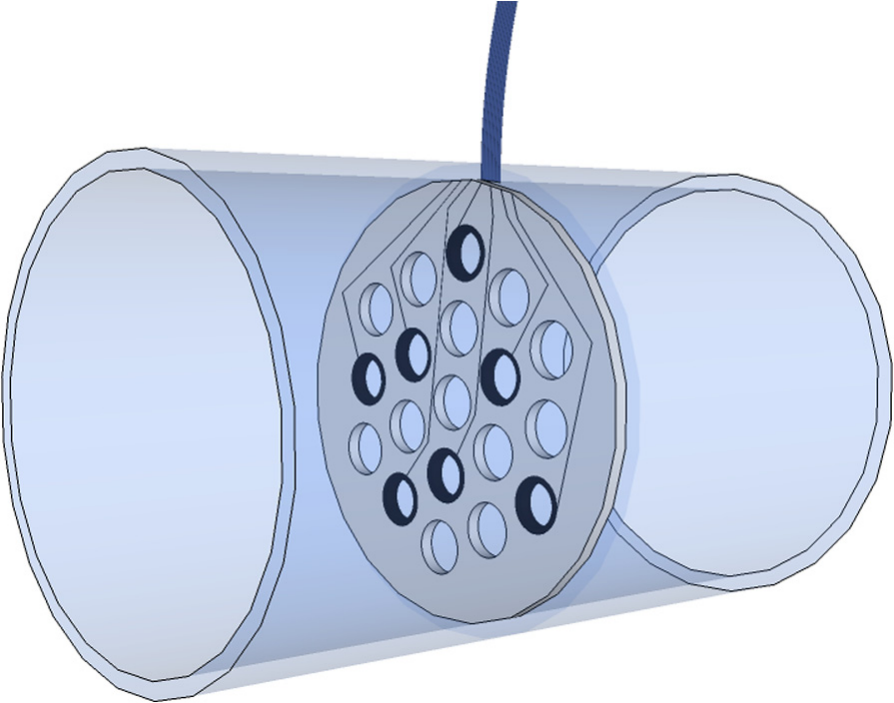
**Sieve electrode.** Illustration of a sieve electrode where the sensing area is around the holes, even if 100% of the holes do not have a contact.

The regenerative electrodes are probably the most invasive type of neural interfaces. Regenerated axons from transected nerves are guided to grow through these electrodes, thus potentially providing higher selectivity. The sieve electrode is the most known regenerative design, Figure 8. It has been found that sieve designs with small holes and higher transparency produced the best results in a comparative study [82].

Sieve electrodes are implanted by severing the nerve, introducing the electrode between the severed ends, and then guiding nerve growth through the electrode holes via a conduit. No clinical trials in humans had been published at the time of this research, but several experiments have been performed in animal models [82-85]. It has been argued that, although the nerve regeneration is not optimal, it is enough to perform normally. This was concluded from a 30-month study in cats that would correspond to 15 years of human life span [84]. However, Lago *et al.* have found a decline in reinnervation and loss of regenerated fibers after 6 months [86]. Furthermore, it has been found that larger motor and sensory axons were overrun by the smaller ones while growing through the sieve holes, and thus limiting selectivity.

The next level on selectivity and cross-talk reduction is the regenerative microchannel electrode, a combination between sieve and cuff electrodes that better isolates bundles of axons [87]. An important feature of this interface is its tolerance to demanding mechanical handling such as twisting and bending (ultra-compliant). Currently, only animal testing has been done using five evaporated Ti/Au/Ti electrodes of sub-100 nm thickness in polydimethylsiloxane (PDMS). This was a 3.5 mm long and narrow micro-cuff (100*μm*×70*μm*cross section) that demonstrated acceptable SNRs (∼4).

Although these are promising neural interfaces, extensive research is required to prove their safety and long-term stability. Specially due to their traumatic implantation and invasive nature.

### Emerging technologies for neural interfaces

Polymer poly(3,4-ethylenedioxythiophe), known as PEDOT, is an electroconductive polymer that is designed to act as a stable, low-impedance, high-fidelity, bidirectional peripheral nervous system interface for the control of prosthetic devices. PEDOT has been shown to have a very low impedance *in vitro* and the conduction of afferent and efferent nerve action potentials does not appear to deviate from that of an intact nerve *in vivo* (rat model) [88]. Another example of emerging technologies is the use of microfabricated ion-selective membranes [89]. This electrochemical method demonstrated an approximately 40% reduction in the stimulation thresholds in an animal model and promising selectivity. Although promising, these are relatively new technologies that require more evaluation to prove their safety and efficacy.

In this paper, we have focused on biopotential electrodes being placed in the peripheral nervous system, however, some of them can also be used in the central nervous system (e.g. micro-electrode array) as so-called brain computer interfaces (BCI). Recently, a new technique has been developed to better integrate electrodes with the curvilinear surfaces of biological tissue. A bioresorbable substrate of silk fibroin allows the fixation of ultra-thin electrodes and, once reabsorbed, capillary forces hold the electrode in place [90]. The advantages of the more intimate integration of the electrodes in complex curvilinear surfaces are obvious, i.e. improved contact and stress minimization. Although it was tested for BCIs, it could also used in the peripheral nervous system.

## Discussion

### Surface Electrodes (SEs) vs. Implantable Electrodes (IEs)

Currently, the most advanced commercial robotic prostheses^a^ are limited to the proportional control (speed and strength) of basic movements (i.e. opening and closing the hand). They all use surface electrodes to read myoelectric signals (MES) from relatively strong muscle contractions, which is inefficient and unnatural for the patient, thereby complicating prosthesis acceptance [91]. Several solutions have been implemented to cope with the poor myoelectric signals provided by surface electrodes; for instance, in order to control two different devices (e.g. hand and wrist), the fast contraction of all the muscles involved is used to switch from one device to the other. Although practical, an approach of this kind is still unnatural and cumbersome.

The lack of classification algorithms [11] and control systems [92] was once the main issue in accomplishing an advanced prosthetic control. Nowadays, several researchers have demonstrated that it is possible to identify finger and hand positioning using a variety of pattern recognition algorithms, such as artificial neural networks (ANNs), support vector machine (SVM), hidden Markov models (HMM), wavelets and so on [91-98].

Now that pattern recognition algorithms and hardware for real-time control are available, the long-term stability of the biopotential signals has become the major issue. Signal stability is strongly related to the acquisition method and source. The following considerations must be kept in mind when designing an acquisition strategy for natural control. 

1. **The control source.** The control origin has an impact on how natural it is for the patient to produce the information required for a given movement. In other words, the control source must be physiologically appropriate in order to be natural for the user.

2. **The information content.** This relates to the information required for the simultaneous control of different degrees of freedom. Simultaneous control is an essential feature when it comes to mimicking the natural physiological systems.

3. **The long-term consistency of the signals.** Although the PRAs and control systems are designed to be robust and tolerate a certain amount of noise, they are still dependent on the consistency of the inputs [10]. Numerous failures, such as low responsiveness and false predictions, tend to frustrate the user [99].

The following is a non-exhaustive list of the issues found when implementing more advanced prosthetic control based on PRAs. They all conflict with at least one of the subsequent considerations, especially the long-term consistency of the signals. 

· Signals recorded using SEs change dramatically with the environmental conditions, i.e. sweating.

· SEs cannot remain in place indefinitely due to skin-related issues and they therefore require daily placement.

· SEs cannot be placed consistently in the same location after removing the prosthesis. A different placement of the electrodes usually requires the re-training of the PRA, or recalibration.

· Artifacts are very easily generated when using SEs due to limb movement and electrode liftoff.

· Patients need to have a minimum level of myoelectric signals to become candidates for using myoelectric prostheses. This is not always the case, depending on the amputation level and the muscle surface left for the electrode placement.

· A wide limb surface area needs to be covered to have enough control signals.

· Control sites are insufficient in patients with high amputation levels. Moreover, as a rule, the few sites available are physiologically inappropriate.

· Muscle imbalance could be created if the electrodes are improperly placed, resulting in some muscles being more exercised than others. In the long term, this will cause the signals from the larger muscles to mask those from the smaller ones. Muscle imbalance can also cause prosthesis socket instability [11].

· The lack of natural feedback, or any feedback apart from visual, complicates the use and acceptance of the prosthetic device.

It has been suggested that the long-term stability of bioelectric signals required for a precise control of several degrees of freedom, is the major issue in robotic prostheses [3,10,25,26,99]. By reviewing the previous list, it is reasonable to argue that this can be mainly attributed to the use of SEs. As mentioned before, the currently available commercial prostheses, and most of the research on prosthetic control, use SEs [10], mainly because they are easy to manufacture and non-invasive. On the other hand, the way in which the nature of SEs inherently impacts the problems mentioned above is obvious. This raises the question of how much better the IEs are in comparison to their surface counterparts. Selecting between SEs and IEs involves different trade-offs, such as the global sensitivity and specificity of the recorded bioelectric potentials. Table 1 summarizes some of the most important differences.

**Table 1:** Comparison between surface and implantable electrodes

		
	**Surface Electrodes**	**Implantable Electrodes**
**Installation**	Stabilization over the skin is necessary	A clinical implantation is required
**Stability**	Highly environmentally dependent (i.e. sweaty or dry skin)	Long-term stable impedance after tissue encapsulation
**Recording field**	Unable to record information from all the muscles in the stump due to muscle superposition	Recordings from individual motor units are possible, as well as direct recordings from nerves
**Information**	Provide a more general spatial characterization of the action potential distribution	Allow more sites for control, reduce cross-talk and provide access to information from nerves that innervated missing muscles
**Chronic use**	Skin and stabilization problems prevent a long placement	Different muscle- and nerve-based electrodes have been chronically implanted in humans

It has been widely suggested that implantable electrodes acquire signals of better quality [17,100], but, when it comes to improvements in pattern recognition, Hargrove *et al.* did not find any significant difference between using 16 down to 4 SEs or 6 intramuscular electrodes while classifying 10 different types of isometric contraction [101]. Farrell and Weir also conclude that the selection between IEs and SEs should be made according to clinical considerations. This was argued after performing a more in-depth study comparing surface and intramuscular electrodes. In this study, key muscles were targeted and compared with a symmetric electrode distribution [102]. It is worth noting that the latter studies were performed using intramuscular electrodes and the question of whether using implanted *nerve-based electrodes* will improve pattern classification is still unanswered. It is possible to argue that the information contained in the nerves that innervated the missing muscles could make a difference in classification performance. Furthermore, IEs, specifically nerve-based electrodes, would provide more physiologically appropriate information, and would also be suitable for a closed-loop control through stimulation of the afferent nerves (feedback).

Independently of the latter assumptions and considering that both types of electrode are able to perform the same control, IEs still solve most of the practical problems associated with SEs. It has been shown that by using implanted electrodes, the signals are more consistent over time and less affected by surrounding noise [103]. Furthermore, it has been reported that patients using multifunctional prostheses with SEs experience fatigue after 5 to 30 min of continuous use [104]. This can be attributed to the large muscle effort that is required to produce suitable myoelectric signals. This effort is considerably less when using IEs, as there is no skin and fat between the electrode and the source. Fatigue is not only unpleasant for the patient, it also complicates pattern recognition.

Several authors have found that at least 4 SEs are required to reach a good degree of classification accuracy when using PRAs [101,102,105]. In the case of intramuscular electrodes, it was suggested that more than 4 electrodes are necessary, since the recording is more localized [102]. It is noteworthy that intramuscular electrodes are more selective than extramuscular ones (such as epimysial) and for this reason, the question of whether more extramuscular electrodes are required for the same purpose still needs to be investigated.

The latter exemplifies the tradeoff between the global sensitivity and specificity of the recorded signals. In a direct control scheme where one signal is paired to one action, a localized measurement is more desirable, and provided that there are as many control sites as movements to control, this would be the most straightforward path to achieve simultaneous control.

Simultaneous control of 2 degrees of freedom using the direct control scheme was first demonstrated by Kuiken *et al.*[106]. This was only possible due to the Targeted Muscle Reinnervation (TMR) procedure which resulted in new and independent control sites. Unfortunately, even in TMR patients with augmented myoelectric control possibilities, it is not always possible to isolate MES satisfactorily from surface recordings. This is thus in favor of the development of implantable MBEs.

Although not as extensive as in sequential control, PRAs and biologically inspired algorithms have been used to predict simultaneous limb movements since 1973 by Herberts *et al.*[107], and more recently by Jiang *et al.*[108], and Muceli *et al.*[109]. Again, these were laboratory experiments using surface electrodes that could potentially be translate into long-term implementations using implantable electrodes.

Kilgore *et al.* have shown that the durability of implantable electrodes, leads and connectors is no longer a major concern in implantable neuroprostheses. They looked at 238 electrodes implanted from 3 to 16 years, where only 3 reported failure. Furthermore, most of the leads crossed different joints, which increased the level of mechanical stress, and yet a 98.7% probability of being functional after 16 years was still reached, even in contracting skeletal muscles [22]. Although these were used in neurostimulators, the safety, biocompatibility and recording performance can be seamlessly applied to prosthetic control. Prosthetic control, however, faces the issue of permanent communication with an outer-body device. Neurostimulators, on the other hand, can be completely implanted and rarely require communication with the outside.

### Muscle-based Electrodes (MBEs) vs. Nerve-based Electrodes (NBEs)

In the case of an amputation, most of the muscles required for dexterity are lost. However, the axons that used to innervate those muscles remain indefinitely viable on the nerves close to the stump [110]. It has been suggested that nerve signals could be used to control prostheses when it was found that the modulation of severed efferent neurons was still related to identified movements [111]. A feasibility study from 1982 conducted in the median, ulnar and radial nerve of a below-elbow amputee supported the latter theory [112]. A more recent study by Jia *et al.* showed that, even after 28 months of being amputated, a patient was able to generate neural activity related to the phantom limb movement [113]. These signals were enough to control simple movements in a robotic prosthesis, although the patient only trained for 2 weeks prior to the experiment. Kuiken *et al.*’s work on TMR provides another strong argument in favor of the viability of nerves after amputation [114].

Currently, as many as 3 different neuroelectric signals, both efferent and afferent, have been differentiated using pattern recognition algorithms. Micera *et al.* classified 3 movements to control a robotic hand using 4 intrafascicular electrodes (tf-LIFE4) [71] and Raspopovic *et al.* showed that the identification of 3 afferent stimuli is possible using single-channel cuff electrodes [43].

Theoretically, NBEs are very attractive because of the large amount of information they would make it possible to retrieve. Signals to several muscles can be obtained from a single nerve and several MBEs would therefore be necessary to produce the same information as one NBE with several contacts. It is noteworthy that the latter would not apply if the electrodes were placed in single fascicles. In both cases, NBEs could still provide information from missing muscles, information that could even be recorded from individual fascicles [65]. Furthermore, it has been observed that neuroelectric signals (NES) show greater shape regularity than the MES [36,115]. This facilitates pattern recognition and thereby improves the stability of the control system.

Since the MES are in the order of mV and the NES are in the order of *μV*, the former are easier to record. Another complication for NBEs is that the MES of surrounding muscles can mask the NES, further complicating their recording. On the other hand, biosignals peak in different spectra (MES 200 Hz, NES 2,000 Hz), making filtering feasible [17]. In some cases, a band-pass filter from 1,000 Hz to 10,000 Hz or a high-roll-off high-pass filter have been sufficient to cut off the MES [31].

The variable position of the MBEs in relation to the muscle contraction results in a position-dependent signal [18]. The latter is not an issue in NBEs, which are also more efficient for stimulation purposes because they use less current and have better selectivity than the MBEs [116]. Furthermore, NBEs eliminate the problems of mechanical stress that plague MBEs, making NBEs more stable over time and giving both the electrode and the leads a longer life expectancy [28].

Finally, NBEs are inherently more suitable for providing feedback that is perceived in a more natural way by the patient. Closed-loop control using different NBEs has been reported in animal models [115] and human patients [40,62].

### Suitable IEs for long-term and natural prosthetic control

The *intra*-type implantable electrodes (i.e. intraneural) break and damage the tissue where they are implanted and therefore have a higher associated risk than the *extra*-type [51]. The need for devices such as the ACTIN resulted from the degradation of the long-term recording qualities of intrafascicular electrodes as a consequence of the fibrous encapsulation that takes place [66]. While it has been suggested that encapsulation is beneficial in epimysial and cuff electrodes [18,29], it is a major issue in intraneural electrodes [72]. This is mainly due the higher impact of attenuation on the smaller signals. Nevertheless, most of the published research on electrodes with direct application to robotic prostheses has used this more invasive designs [26,70,71,102,113]. On the clinical side, however, *extra*-type electrodes such as the epimysial and cuff, have proved their safety and functionality for several years in different applications [19-22,31-34,40-42,52]. It is worth noting that a screened epimysial electrode design as in Figure ??, with a short inter-electrode distance, would result in a very localized sensitivity. This would practically be equivalent to the use an intramuscular electrode for direct control purposes. Furthermore, low spatial resolution (high selectivity), also implies low information content. Selectivity and global sensitivity both highly depend on the electrode design. Therefore, electrodes must be carefully selected considering the available control sites and the desired control strategy.

It has been shown that neural interfaces can provide enough information to allow PRAs to identify different movements [43,71]. It seems fair to argue that the combination of implanted NBEs and MBEs has the potential to solve the 3 main problems mentioned in the surface vs implantable electrodes discussion. 

1. **The control source.** When using muscle- and nerve-based interfaces, the origin of the control signals is the same as that of an intact limb. Especially with neural interfaces where the information would be physiologically more appropriate even in high-level amputees. Feedback would be also transmitted directly in the remaining natural conductors.

2. **The information content.** Nerves contain all the information required for natural control. Arguments in favor of their viability after amputation have been given in this work, as well as citations of different experiments in which recordings and stimulations had been achieved. MBEs could well complement the control system increasing the number of sites for control. In some cases such as in low trans-radial amputees, MBEs alone provide a considerable amount of control sites.

3. **The long-term consistency of the signals.** Implantable electrodes, especially the epimysial and cuff electrodes, have been shown to be stable in long-term implementations where the consistency of the electrode impedance has been observed. This feature directly relates to the stability of bio-electric signals.

In cases in which suitable nerve recordings for pattern recognition cannot be obtained due to technical limitations, it should still be possible to use epimysial electrodes to achieve the stability required for long-term implementation. Despite the fact that information from muscles might not be sufficient to achieve dexterity, since most of the required muscles are lost in an amputee, an approach of this kind will still dramatically increase the functionality of the prosthesis by increasing the number of movements and allowing simultaneous control. A simple control scheme pairing single movements with individual, but well isolated myoelectric signals, would be enough to produce simultaneous movements. Finally, the combination of nerve and muscle recordings could ultimately be used to achieve long-term stable and natural prosthetic control.

It is worth mentioning that electrode technology continues to progress and new materials, coatings or surface treatments such as PEDOT [88], silk fibroin [90] and ion-selective membranes [89] could eventually replace or be applied to standard epimysial and cuff electrodes. These are just a few examples of potential future improvements that could eventually enter the realm of clinical applications.

## Conclusions

We have presented evidence suggesting that both muscle- and nerve-based implantable electrodes, if properly located and implanted, are a viable solution for the long-term implementation of natural prosthetic control. NBEs have the potential to provide information from missing muscles to achieve dexterity and, although we have cited different experiments proving the feasibility of nerve recordings, MBEs are still required to complement the control system - mainly for the practical convenience of higher amplitudes and locations that ease signal recording.

This IE solution, however, is accompanied by a major new issue, namely, access to the signals from outside the body. The majority of experiments demonstrating the most advanced prosthetic control using implantable electrodes have relied on percutaneous communication [43,62,63,70,71]. A long-term implementation, however, requires the elimination of percutaneous wires [36,41]. The percutaneous passage of wires must be avoided completely because it provides a path for viruses and bacteria [17]. A promising effort in IEs by Weir *et al.* includes the IMES, a wireless myoelectric sensor that uses RF transmission to avoid the percutaneous connection [24]. On the neural side, the UEA group is also developing a wireless strategy [74]. Several problems are, however, associated with the use of RF in this application. The continuous use of the prosthesis requires the constant transmission of information, thereby increasing the power requirements. One dynamic biocompatibility issue in relation to constant transmission is the over-heating of surrounding tissue. According to the international standard ISO14708-3:2008, Implants for surgery - Active implantable medical devices, no outer surface temperature of an implantable device should be greater than 2°C above the normal surrounding body temperature. Furthermore, the changing device orientation relative to the coil as caused by limb movements is a potential source of malfunction that are still to be addressed [26].

Finally, it can be concluded that the use of IEs as a solution for the long-term stable and natural control of robotic prosthetics depends on the method that is used to enable the IEs to communicate with the artificial limb. When communication with the electrodes from outside the body is not guaranteed, the long-term implementation is therefore compromised.

## Endnote

^a^e.g. Utah Arm (Motion Control, Inc., USA), Boston Arm (Liberating Technologies, Inc. USA), SensorHand DMC (Otto Bock, Germany), i-LIMB (Touch Bionics Inc, UK) and Bebionic (RSLSteeper, UK)

## Abbreviations

ACTIN, Actuated intraneural interface; BCI, Brain computer interface; CNS, Central nervous system; EEI, Electrode-electrolyte interface; EMG, Electromyography; ENG, Neurography; FES, Functional electrical stimulation; FINE, Flat interface nerve electrode; IEs, Implantable electrodes; IMES, Implantable myoelectric sensors; LIFE, Longitudinal intrafascicular electrodes; MBEs, Muscle-base electrodes; MEAs, Micro-electrode arrays; MES, MyoElectric Signals; MEMS, Micro-electro-mechanical systems; NBEs, Nerve-base electrodes; NES, NeuroElectric Signals; PEDOT, Polymer poly(3,4-ethylenedioxythiophe); PolyLIFE, Polymer-based longitudinal intrafascicular electrodes; PRAs, Pattern recognition algorithms; SEs, Surface electrodes; SNR, Signal-to-noise ratio; TIME, Transverse intrafascicular multichannel electrode; TMR, Targeted muscle reinnervation; UEA, Utah electrode array.

## Competing interests

In addition to governmental institutions, this work was partially funded by Integrum AB which is currently investing in the advanced control of robotic prostheses.

## Author’s contributions

MOC performed the review, analysis and drafted the manuscript. RB, BH and JD supervised this research and revised the manuscript. All the authors have read and approved the final manuscript.
